# A Markov Chain Model for Determining the Optimal Time to Move Pregnant Cows to Individual Calving Pens

**DOI:** 10.3390/s23198141

**Published:** 2023-09-28

**Authors:** Cho Nilar Phyo, Pyke Tin, Thi Thi Zin

**Affiliations:** 1 Interdisciplinary Graduate School of Agriculture and Engineering, University of Miyazaki, Miyazaki 889-2192, Japan; chonilarphyo@gmail.com; 2 Graduate School of Engineering, University of Miyazaki, Miyazaki 889-2192, Japan; pyketin11@gmail.com

**Keywords:** video monitoring for periparturient cows, optimal time scheduling, Markov Chain Model, individual calving pens, absorbing barriers, real-life experiments

## Abstract

The use of individual calving pens in modern farming is widely recognized as a good practice for promoting good animal welfare during parturition. However, determining the optimal time to move a pregnant cow to a calving pen can be a management challenge. Moving cows too early may result in prolonged occupancy of the pen, while moving them too late may increase the risk of calving complications and production-related diseases. In this paper, a simple random walk type Markov Chain Model to predict the optimal time for moving periparturient cows to individual calving pens was proposed. Behavior changes such as lying time, standing time, and rumination time were analyzed using a video monitoring system, and we formulated these changes as the states of a Markov Chain with an absorbing barrier. The model showed that the first time entering an absorbing state was the optimal time for a pregnant cow to be moved to a calving pen. The proposed method was validated through a series of experiments in a real-life dairy farm, showing promising results with high accuracy.

## 1. Introduction

In the dairy farming industry, it is common practice to move pregnant cows to an individual calving pen when they show signs of calving. However, the effects of this practice on cow behavior during labor and the progress of the calving process are not clear. Most existing methods for moving pregnant cows to an individual calving pen are based on empirical data analysis and three movement strategies which include:

1. The just-in-time calving pen strategy, where within hours of giving birth, cows are moved to the calving pen;
2. The short-stay calving pen strategy, where cows are moved to the calving pen less than two days before calving;
3. The long-stay calving pen strategy, where cows are moved into the calving pen more than seven to 14 days before calving;

The just-in-time strategy (strategy 1) is primarily used by larger herds with staff members primarily responsible for transition cows and calving. This strategy requires around-the-clock supervision of the transition group, and staff must be trained to identify the stages of labor. Moving cows with a waterbag or feet showing to the calving pen can significantly reduce the risk of stillbirth. However, without adequate training and time to walk the transition yards, calves may be born outside of the calving pen. Short-stay strategies (strategy 2) allow cows to be moved within two days of calving, reducing social stress during regrouping. These strategies tend to be used in smaller herds and require excellent timing in the identification of calving and a group calving pen to avoid the prolonged isolation of individual cows. Long-stay strategies (strategy 3) involve moving cows into the calving area more than seven days before calving, but predicting an individual cow’s calving time is virtually impossible.

Instead, a group approach can be taken for larger herds, where a group of cows is moved from the dry cow group to the calving pen each week. The group calving pen has sufficient capacity to accommodate one week’s worth of calving cows, with sufficient separate calving pens to accommodate each group until they all calve. Some studies suggest that moving a cow during stage 1 can prolong calving and cause complications [1,2]. However, some researchers argue that predicting calving time can lead to developing a model for inferring the optimal time to move cows to calving pens [3,4]. It is also important to note that theoretical aspects should be considered when looking at such findings.

To address this issue, this paper proposed a simple random walk type Markov Chain Model for predicting the optimal time for moving periparturient cows to individual calving pens. The remaining parts of the paper are structured as follows: Section 2 provides an overview of related works in the field. Section 3 presents the materials and methods used in the proposed problem architecture, followed by the experimental works and results in Section 4. Statistical analysis and discussions of the results are presented in Section 5. Finally, the concluding remarks are provided in Section 6.

## 2. Some Related Works

In the early stages of this study, we delved into the existing methods that were proposed to address the issue at hand. Three primary approaches have surfaced in previous research [1,5,6]. These methods encompass distinct strategies for managing cow movement during the critical calving period, each with its own set of advantages and disadvantages. Method 1, known as the ‘just-in-time calving pen’ method, involves swiftly relocating cows to dedicated calving pens within hours of the impending birth. Studies [1,5,6] have indicated those cows moved using this approach experienced certain benefits, such as reduced labor duration during the second stage of calving, compared to the alternative methods (Methods 2 and 3).

However, it is worth noting that contrasting findings exist in the literature. In some cases, where cows were moved to maternity pens just before calving (Method 1), there were no significant differences in postural changes during the hour preceding calving when compared to other categories. Nonetheless, these observations may have been con-strained by the limitations of small sample sizes.

Intriguingly, only one study [7] has explored the impact of transitioning cows from a group setting to an individual maternity pen on their overall health outcomes. In scenarios where producers opt for the practice of relocating cows to individual maternity pens just before calving, there remains a critical question: “Is there optimal timing for this movement to occur?” This question serves as the focal point of our research, as we aim to shed light on the optimal timing for this pivotal transition.

In addition, several studies have been conducted on the management of dairy cows during the transition period, including the timing for moving a pregnant cow to an individual calving pen. Studies have found that cows may be moved before labor, during early stage one labor, or during late stage one labor, depending on the signs they exhibit, and short-stay strategy is preferable, but more experimental work is needed to confirm their findings [1]. Cows moved during late stage one labor have been found to have longer labors, but no longer contractions compared to cows moved during other stages [5,6]. Despite the lack of clear data, many farms continue to move cows to individual maternity pens just before calving based on physical and behavioral cues exhibited by cows before and during labor. However, the timing of these cues can vary greatly, making it difficult to determine when a cow should be moved to a calving pen [8].

A survey paper indicates that in most countries and regions, dairy cows calve in group pens or individual pens to which they are moved with signs of imminent calving. Moreover, moving cows when they show signs of imminent calving may increase the duration of labor [9]. However, recent research suggests that calving facilities offer no opportunity for cows to seek seclusion during calving, which is a behavior observed in wild and domesticated ungulates before calving [7,10,11]. The discrepancy between current practice and recommendations places dairy cow welfare at risk and calls for further research and development in calving management systems [12]. A study has also observed that socially dominant cows are more likely to calve in an individual pen, while the presence of newly born calves to other cows may also influence calving behavior [13]. Therefore, some researchers proposed a queueing inventory system for calving management with a different approach in [14,15,16].

The housing and management of the calving section, such as low space allowances in group calving pens, may compromise the isolation-seeking behavior of cows before calving [17]. It is worth mentioning some results and findings of [17] that animals increased their locomotor behavior from 24 to 2 h before calving and decreased their locomotor behavior from 2 to 0 h before calving. The increasement in locomotor behavior before calving has been driven by the motivation of finding a desirable calving site or separating from other cows due to discomfort associated with giving birth. On the other hand, separation behavior refers to the way in which a cow interacts with other cows or animals in its environment. In the context of a pregnant cow near calving time, separation behavior is an indication that the cow is preparing to give birth. For example, she may begin to isolate herself from the rest of herd or become more aggressive to other cows. These behaviors are also useful to predict the time to calving events.

Farmers believe that the timing of relocation can affect calving behavior, and according to “Veterinary Reproduction and Obstetrics”, cows should not be moved during late stage one labor, and this causes stage two labor to prolong by 30 min [3]. The decision of when to move cows to an individual maternity pen is often made based on predicting the time until the calving event [4]. Experts suggest that the best time to move a cow to the pen is 12 to 24 h before the predicted time or the expected due date [17]. For predicting the optimal time for moving pregnant cows to calving pens, a simple Markov Chain Model based on a random walk is proposed in this paper. The proposed approach can offer a novel way to predict calving time, which may lead to improved management strategies for dairy cows during the transition period.

## 3. Materials and Methods

**Figure 1:**
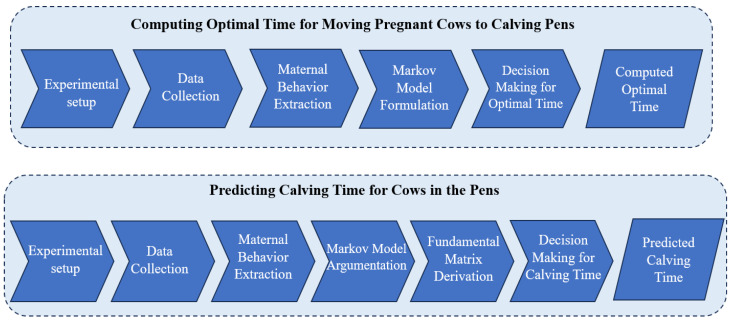
Overview of the proposed system.

The proposed system consists of two subsystems: (i) computing the optimal time for moving pregnant cows to calving pens and (ii) predicting the calving time for cows in the pens. This study involved an experimental setup, data collection, the extraction of maternal behavior, the formulation of the Markov Model, and decision-making processes, as shown in Figure 1.

### 3.1. Subsystem 1: Computing the Optimal Time for Moving Pregnant Cows to Calving Pens

#### 3.1.1. Experimental Setup

During the experiment, a video monitoring system was installed to cover the entire area where the calving cows were kept. Video recordings were continuously performed during both the day and night to capture the activities of calving cows before they were moved to the calving pens. The video recordings took place starting two weeks before the expected calving date.

#### 3.1.2. Observation of Behaviors

Cattle behaviors during the calving period were systematically classified into six dis-tinct types: feeding, moving, standing resting, lying resting, stand rumination, and lie rumination. This classification was achieved through meticulous human annotation and observation. Trained observers continuously monitored the cows and recorded their behaviors at hourly intervals until the cow was eventually relocated to the calving pen. While the long-term goal of this study is to automate the extraction of these behaviors using advanced machine learning techniques, it is important to note that this automation process has not been fully implemented at this stage. Consequently, the primary focus of this research remains on the manual extraction and thorough documentation of these behaviors.

#### 3.1.3. Formulation of State Sequence

The observed behaviors (minutes) were continuously recorded and hourly organized to form Multivariate Time Series (MTS) data. Then, we used them to formulate the state sequence based on Mahalanobis distance. Multivariate Time Series (MTS) refers to a collection of time-ordered data points, where each data point consists of multiple variables. Those time series data are commonly used in various domains, such as finance, environmental monitoring, and industrial processes. Mahalanobis distance is a statistical metric introduced by P. C. Mahalanobis in 1936 [18] to quantify the distance between a point and a distribution. This distance metric is highly effective for handling multivariate data and has been applied in various fields, including multivariate anomaly detection, classification on highly imbalanced datasets, and one class classification. The formal definition of Mahalanobis distance between two vectors *x*(*A*) and *x*(*B*) can be defined as

(1) $$d\;\left(Mahalanobis\right)=\sqrt{\begin{array}{ccccc} {\left[(x(B)-x(A))\right]}^{T} & * & C^{-1} & * & \left[x(B)-x(A)\right] \end{array}}$$

where *C* is the sample covariance matrix. Notably, *Mahalanobis* distance is unitless and depends on a positive semi definite (PSD) matrix *C*. Its key advantages include its ability to account for correlations between different variables, leading to more accurate relationships in multivariate time series analysis. Additionally, its scale does not impact the performance of classification or clustering for MTS, making it a reliable local distance metric for comparing multivariate time series data.

The continuously recorded observed behaviors are described as column {*z*_1_, *z*_2_, …, *z*_7_}. The correlation between observed behaviors is expressed by covariance matrix Σ, i.e.,

(2) $$\Sigma =\left[C_{ij}\right],\;\mathrm{for}\;i,\;j=1,\;2,\;\ldots .,6,\;\mathrm{where}\;C_{ij}=Cov(z_{i},z_{j})=E[\begin{array}{cc} \left(z_{i}-\mu_{i}\right) & \left(z_{j}-\mu_{j}\right) \end{array}]$$

The *Mahalanobis* distance between data points *x* and *y* is

(3) $$MD=\sqrt{\left[\begin{array}{cc} {\left(x-y\right)}^{T} & \begin{array}{cc} {\left(\sum \right)}^{-1} & \left(x-y\right) \end{array} \end{array}\right]}$$

Then, each row will be labeled as state number *j* if the minimum value in *MD* corresponds to *z_j_* for *j* = 1, 2, …7. Let the resulting state sequence be

(4) $$\left\{s_{1},\;s_{1},\;s_{3},\;s_{5},\;s_{4},\;s_{4},\;s_{1},\;s_{6}\ldots \ldots ..\right\}$$

#### 3.1.4. Creation of Co-Occurrence Matrix and Markov Chain Model

The co-occurrence matrix and probability matrix of the Markov Chain Model are created by using the state sequence described in Equation (4). There are seven states in the sequence of Equation (4); thus, 6 × 6 co-occurrence matrix *C* and transition probability matrix *P* can be established as follows:

(5) $$C=\begin{array}{cc} & \begin{array}{cccccc} s_{1} & s_{2} & s_{3} & s_{4} & s_{5} & s_{6} \end{array}\\ \begin{array}{c} s_{1}\\ s_{2}\\ s_{3}\\ s_{4}\\ s_{5}\\ s_{6} \end{array} & \left[\begin{array}{cccccc} c_{11} & c_{12} & c_{13} & c_{14} & c_{15} & c_{16}\\ c_{21} & c_{22} & c_{23} & c_{24} & c_{25} & c_{26}\\ c_{31} & c_{32} & c_{33} & c_{34} & c_{35} & c_{36}\\ c_{41} & c_{42} & c_{43} & c_{44} & c_{45} & c_{46}\\ c_{51} & c_{52} & c_{53} & c_{54} & c_{55} & c_{56}\\ c_{61} & c_{62} & c_{63} & c_{64} & c_{65} & c_{66} \end{array}\right] \end{array}P=\left[\begin{array}{cccccc} p_{11} & p_{12} & p_{13} & p_{14} & p_{15} & p_{16}\\ p_{21} & p_{22} & p_{23} & p_{24} & p_{25} & p_{26}\\ p_{31} & p_{32} & p_{33} & p_{34} & p_{35} & p_{36}\\ p_{41} & p_{42} & p_{43} & p_{44} & p_{45} & p_{46}\\ p_{51} & p_{52} & p_{53} & p_{54} & p_{55} & p_{56}\\ p_{61} & p_{62} & p_{63} & p_{64} & p_{65} & p_{66} \end{array}\right]$$

where *c_ij_* is the number of pairs (*s_i_*, *s_j_*) for *i*, *j* = 1, 2, …6 and $p_{ij}=c_{ij}/\sum_{i=1}^{i=6}[c_{ij}]$.

The sum of the row probabilities is equal to one since each health state is independent of the other and an animal must move to one of the seven states. The diagonals represent the probability of staying in the same state. A state is considered absorbing when the probability of leaving a state is zero. In this study, the absorbing state is moving to the individual calving pen. When the moving event occurs, further investigations shall be stopped in subsystem 1, since it is attempting to predict the time to move to the calving pen. Thus, the seven states are known as transient states of the Markov Chain, since, from every state, the system can move to another state independently. In this Markov Chain, the time unit is hours.

#### 3.1.5. Prediction Procedure for Time to Move to Calving Pen

The calving time prediction is performed by using the Markov Chain Model developed in previous sections. In order to do so, the calving event is augmented as an absorbing state of the four-state Markov Chain Model. Since the proposed system deals with the prediction of time to move, the problem will end once the prediction is completed. Therefore, the calving state is assumed to be an absorbing state. That means that a cow can go from any of the other four states to the calving state, but once the system enters the calving state, it will stay there. This reduces the probability of transition from absorbing state to absorbing state, which is 1, and the transition probability from the absorbing state to any other state is 0. The six-state Markov Chain Model described in Equation (5) is transformed into an Augmented Markov Chain Model A of five states by adding the absorbing (calving) state, as shown in Equation (6). *MTC* refers the state of moving to a calving pen; *α_i_* for *i* = 1, 2, …,6 probabilities of absorbing from state *i*, respectively. Due to the properties of the transition probability matrix, each row sum in the augmented matrix must be 1. To clarify this notation, the subscript notation *α_ij_* is used to represent the elements in the augmented matrix. Durations in transient states and expected calving time can be estimated from Markov models using the matrix solution and Monte Carlo simulation.

(6) $$A=\begin{array}{cc} & \begin{array}{cccccc} s_{1} & s_{2} & s_{3} & s_{4} & s_{5} & \begin{array}{cc} s_{6} & MTC \end{array} \end{array}\\ \begin{array}{c} s_{1}\\ s_{2}\\ s_{3}\\ s_{4}\\ s_{5}\\ \begin{array}{c} s_{6}\\ MTC \end{array} \end{array} & \left[\begin{array}{cccccc} \alpha_{11} & \alpha_{12} & \alpha_{13} & \alpha_{14} & \alpha_{15} & \begin{array}{cc} \alpha_{16} & \alpha_{1} \end{array}\\ \alpha_{21} & \alpha_{22} & \alpha_{23} & \alpha_{24} & \alpha_{25} & \begin{array}{cc} \alpha_{26} & \alpha_{2} \end{array}\\ \alpha_{31} & \alpha_{32} & \alpha_{33} & \alpha_{34} & \alpha_{35} & \begin{array}{cc} \alpha_{36} & \alpha_{3} \end{array}\\ \alpha_{41} & \alpha_{42} & \alpha_{43} & \alpha_{44} & \alpha_{45} & \begin{array}{cc} \alpha_{46} & \alpha_{4} \end{array}\\ \alpha_{51} & \alpha_{52} & \alpha_{53} & \alpha_{54} & \alpha_{55} & \begin{array}{cc} \alpha_{56} & \alpha_{5} \end{array}\\ \begin{array}{c} \alpha_{61}\\ 0 \end{array} & \begin{array}{c} \alpha_{62}\\ 0 \end{array} & \begin{array}{c} \alpha_{63}\\ 0 \end{array} & \begin{array}{c} \alpha_{64}\\ 0 \end{array} & \begin{array}{c} \alpha_{65}\\ 0 \end{array} & \begin{array}{cc} \begin{array}{c} \alpha_{66}\\ 0 \end{array} & \begin{array}{c} \alpha_{6}\\ 1 \end{array} \end{array} \end{array}\right] \end{array}$$

#### 3.1.6. Fundamental Matrix Solution

The matrix solution provides an exact solution of the time spent in each state, restricted to time-homogeneous Markov chains and being conditional on the entry state in which an individual enters the model. The transition probability matrix of a chain contains absorbing states and is divided into four sections, as described in Table 1: *Q* contains transition probabilities between transient states; *R* is the probabilities between transient states and absorbing states; *O* represents a zero matrix; and *I* means an identity matrix.

In the proposed Augmented Markov Model case, *Q*, *R*, *O*, and *I* become as follows:

(7) $$Q=[\begin{array}{cccccc} p_{11} & p_{12} & p_{13} & p_{14} & p_{15} & p_{16}\\ p_{21} & p_{22} & p_{23} & p_{24} & p_{25} & p_{26}\\ p_{31} & p_{32} & p_{33} & p_{34} & p_{35} & p_{36}\\ p_{41} & p_{42} & p_{43} & p_{44} & p_{45} & p_{46}\\ p_{51} & p_{52} & p_{53} & p_{54} & p_{55} & p_{56}\\ p_{61} & p_{62} & p_{63} & p_{64} & p_{65} & p_{66} \end{array}]\;R=\left[\begin{array}{c} \alpha_{1}\\ \alpha_{2}\\ \alpha_{3}\\ \alpha_{4}\\ \alpha_{5}\\ \alpha_{6} \end{array}\right]O=\left[\begin{array}{cccccc} 0 & 0 & 0 & 0 & 0 & 0 \end{array}\right]I=\left[1\right]$$

The iterated multiplication of the augmented matrix *A* yields:

(8) $$A^{2}=\left[\begin{array}{l} Q\;R\\ O\;I \end{array}\right]\times \left[\begin{array}{l} Q\;R\\ O\;I \end{array}\right]=\left[\begin{array}{cc} Q^{2} & \;QR+R\\ O & \;I \end{array}\right]$$

(9) $$A^{3}=\left[\begin{array}{cc} Q^{2} & \;QR+R\\ O & \;I \end{array}\right]\times \left[\begin{array}{l} Q\;R\\ O\;I \end{array}\right]=\left[\begin{array}{cc} Q^{3} & \;Q^{2}R+QR+R\\ O & \;I \end{array}\right]$$

Hence, by the induction of Equation (9),

(10) $$A^{t}=\left[\begin{array}{cc} Q^{t} & \;Q^{t-1}R+Q^{t-2}R+\ldots +R\\ O & \;I \end{array}\right]=\left[\begin{array}{cc} Q^{t} & \;(Q^{t-1}+Q^{t-2}+\ldots +I)\times R\\ O & \;I \end{array}\right]$$

But when *t* tends to infinity, the transient state matrix *Q_t_* will tend to *O* (zero matrix). Then, Equation (10) can be transformed as follows:

(11) $$A^{\infty }=\left[\begin{array}{cc} Q & \;NR\\ O & \;I \end{array}\right]\;N=I+Q+Q^{2}+Q^{3}+\ldots \ldots ={\left(I-Q\right)}^{-1}$$

The matrix *N* = (*I* − *Q*)^−1^ is called the fundamental matrix for the Augmented Markov Chain Model. Let *N*(*i*, *j*) be the element in the row *i* and column *j*. Then, the summation of *N*(*i*, *j*) over *j* is interpreted as the expected number of periods until absorbing occurs (moving to the calving pen). Therefore, the expected time until absorbing (calving state) occurs can be defined as follows:

(12) $$Expected\;Calving\;Time=\sum_{j}\sum_{i}N(i,j)$$

This study formulates an absorbing Markov Chain which comprises six transient states and one absorbing state, resulting in a total of seven states, as ordered in Equation (11). The following definitions are provided to clarify the components involved:

- *Q* is a 6 × 6 matrix that represents the transition probabilities from one transient state to another.
- *NR* is a 6 × 1 matrix that denotes the probabilities of transitioning from a transient state to the absorbing state.
- Matrix 0 is a 1 × 6 matrix consisting of all zeros, symbolizing the impossible transitions from an absorbing state to a transient state.
- *I* is a 1 × 1 identity matrix, representing the impossible transitions between absorbing states, such as remaining in the same absorbing state.

To illustrate the probability of moving from the initial state, denoted as *p*(0), to the absorbing state, the following equation is provided. The probability of absorbing or moving at the expected time is

(13) Probabilty of Absorbtion=p(0)×N×R

### 3.2. Subsystem 2: Computing the Optimal Time for Calving Time

In this section, an absorbing Markov Chain Model will be utilized to predict the optimal calving time. To accomplish this, firstly, the dataset using video sequences captured from the moment a cow enters the calving pens is prepared. As outlined in subsystem 1, six types of cattle activities will be considered. However, a different criterion will be used to label the dominant activity, which involves crossing the mean plus two standard deviations instead of just the mean plus one standard deviation. Then, a new cooccurrence matrix can be obtained from this adjustment.

From the obtained cooccurrence matrix, the corresponding matrix *Q* is deduced, which represents transition probabilities between different cattle activities. This matrix is a fundamental component for this analysis. By deriving matrix (*I* − *Q*), where *I* is the identity matrix, and obtaining its inverse, the optimal calving time can be determined. The resulting fundamental matrix (*I* − *Q*) and its inverse provide valuable insights into the calving process. Summing all entries of the inverse matrix of (*I* − *Q*) allows one to obtain the time at which a calving event is likely to occur.

## 4. Some Experimental Results

Illustrative data were generated to analyze the activities of pregnant cows based on available datasets, specifically focusing on the 10-day period prior to the expected due date. Animal experts have identified several key activities that are frequently observed in cattle nearing the calving time. These activities include feeding, moving, standing in rest, lying in rest, rumination in lying, and rumination in standing. To facilitate the computation process, the activities are defined as states of the system. Each activity is assigned a specific state number: feeding (*s*_1_), moving (*s*_2_), standing in rest (*s*_3_), lying in rest (*s*_4_), rumination in lying (*s*_5_), and rumination in standing (*s*_6_). Sample generated data are presented in Table A1.

In order to identify patterns and make predictions, the mean and standard deviation are calculated for each activity. Generally, as the calving time approaches, the activities tend to occur at a faster pace. This suggests that the number of activities that cross the mean plus one standard deviation can be utilized as an optimal time to move the cow to the calving pen. Additionally, crossing the mean plus two standard deviations can be considered as an indicator of the time to calving event being imminent. By analyzing the generated data and observing the activity trends, the appropriate timing can be effectively determined for moving a pregnant cow to the calving pen. This methodology provides a practical approach to optimizing the management of pregnant cows and ensuring their well-being during the calving process.

From the generated data, the dominant activity for each row can be determined by identifying the activity that is nearest to the mean plus one standard deviation. This allows us to create a sequence of dominant activities for the entire sample dataset. Once the dominant activity sequence has been created, a co-occurrence matrix *C* can be constructed for each individual cow, as described in Equation (A1).

The co-occurrence matrix *C* shows the frequency of transitions between pairs of dominant activities and provides valuable insights into the relationships between different dominant activities patterns of pregnant cows during the pre-calving period. By normalizing the rows of co-occurrence matrix *C*, the Markov Chain Probability Matrix *P*, which can identify the most common transitions, is obtained as Equation (A2) and the dynamics of activity changes in pregnant cows nearing the calving time. This information can be used to further optimize management practices and make informed decisions regarding the optimal time to move cows to calving pens.

### 4.1. Optimal Moving Time to Calving Pen

The move to a calving pen is added as an absorbing state (*s_7_*) in transition probability matrix *P*. Then, an augmented transition matrix *P′* can obtained, as shown in Equation (A3).

From the augmented transition matrix *P′*, the *Q* and *R* matrixes of the Augmented Markov Chain Model can be obtained as Equations (A4) and (A5). Then, matrix *N* which is the inverse matrix (*I* − *Q*) can be derived as Equation (A6). Thus, from the theory developed in Section 3, the sum of all entries gives the expected time of moving the cow to the calving pen, which can be obtained as:

(14) $$Expected\;Calving\;Time=\sum_{j}\sum_{i}N(i,j)=192.57\;\mathrm{hours}=8.02\;\mathrm{days}$$

Therefore, the optimal time for moving to the calving pen can be estimated as 192.57 h = 8.02 days before the expected due date.

### 4.2. Computing the Optimal Time for Calving

The absorbing Markov Chain Model which has been introduced in Section 3.2 will be utilized to predict the optimal calving time. From video sequences captured from the moment of a cow entering into the calving pens, six types of cattle activities and a new criterion of dominant activity, which is the mean plus two standard deviations of each activity, will be applied to establish a new co-occurrence matrix. Then, the corresponding matrix *Q* can be deduced from the new co-occurrence matrix. By deriving the matrix (*I* − *Q*) and obtaining its inverse matrix *N*, as described in Equation (A7), the optimal calving time can be determined by summing all entries of matrix *N*.

In this experiment, the predicted calving time from the starting point is

(15) $$\sum_{j}\sum_{i}N(i,j)=211.67\;\mathrm{hours}$$

Considering that the time required for moving the cattle to the calving pens is 192.57 h, the proposed system can estimate that the cows will calve within 19.1 h after being moved. It is worth noting that according to dairy experts, the optimal time to move a cow to the calving pen should be between 12 and 24 h before the expected due date. Therefore, the predicted time for moving to the calving pen and the subsequent calving time fall within the range of optimal levels. By utilizing the absorbing Markov Chain Model and considering various factors, the predictions about calving time can be informed, leading to more efficient management and care for cattle.

## 5. Statistical Analysis and Discussions

In this paper, the potential of a stochastic and Markov modeling approach has been explored and examined to determine the optimal time for moving a pregnant cow to calving pens, as well as predicting the calving time once the cow is moved. The findings of this research work indicate that the utilization of an absorbing Markov Chain simplifies the problem and allows for the easy calculation of the optimal time for cattle movement. Additionally, the simulated results demonstrate that the optimal time falls within the range of 12 to 24 h before the actual calving time. These findings align with expectations of dairy scientists and hold promising implications for precision dairy farm management systems.

**Figure 2:**
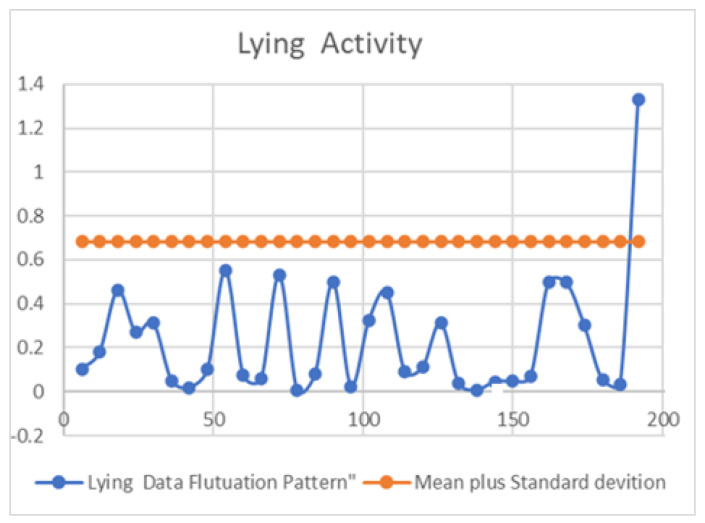
Lying activity data fluctuation pattern with mean plus standard deviation.

**Figure 3:**
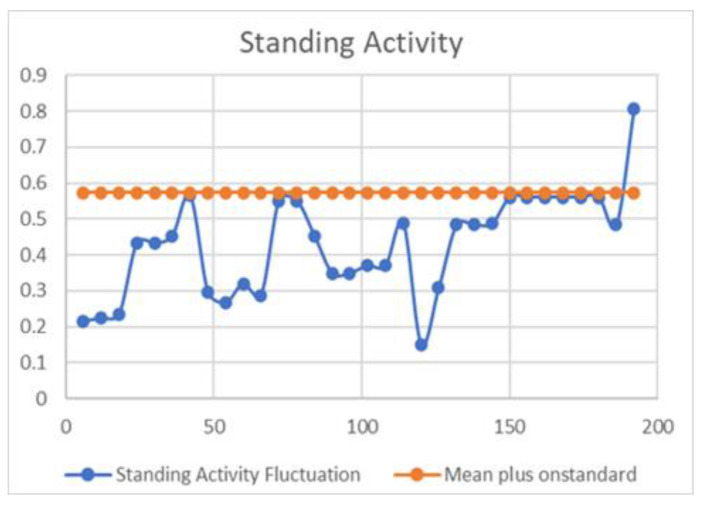
Standing activity data fluctuation pattern with mean plus standard deviation.

**Figure 4:**
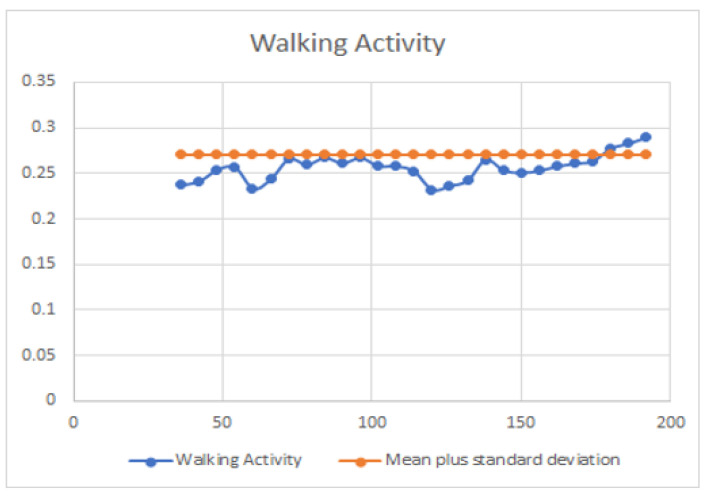
Walking activity data fluctuation pattern with mean plus standard deviation.

However, further research using real-life data and conditions is necessary to confirm and enable practical applications of these findings. It is worth noting that the predictions of moving time and calving time are based on the first few days of data, indicating the potential for accurate outcomes with minimal input. This research is intended to continue in real-life environments to ascertain whether the prediction time and the actual time align closely. From a statistical standpoint, the fluctuations in each activity can support the decision-making process, as depicted in Figure 2, Figure 3 and Figure 4.

Figure 2 illustrates the pattern of lying activity. To analyze this activity, firstly, the mean and standard deviation of the pattern distribution is calculated, and the resulting graphical representation reveals interesting insights. Notably, as cattle approach the calving time, their activity pattern tends to cross the mean plus standard deviation line, as shown in Figure 2.

Figure 3 provides a detailed view of the patterns associated with standing activity. Similarly, the mean and standard deviation for this activity are calculated, and the findings are graphically represented.

Figure 4 delves into the patterns of walking activity. The calculated mean and standard deviation are utilized again to characterize these patterns, which are presented visually in the figure.

These figures serve to elucidate the observations of this study, making it easier to comprehend the relationships between activity patterns and specific events or conditions.

## 6. Conclusions

In this study, a six-state transient and recurrent Markov Chain Model is developed, as well as a seven-state absorbing Markov Chain Model, to determine the optimal time for moving a pregnant cow to calving pens, ensuring that the calving process occurs neither too early nor too late. While Markov Chain Models have been widely applied in various research fields, including engineering, medicine, agriculture, livestock, and animal science, there has been limited development regarding the use of Markov chains in the context of dairy cows moving to calving pens.

This research work explored and examined the applicability of Markov models to dairy cow reproduction management for predicting the optimal time to facilitate a smooth calving event. Six physical behaviors of dairy cows were focused on; however, it is important to include additional activities such as head movements, restlessness, and tail position in future research using the proposed Markov model. In this paper, the parameters for the absorbing state were estimated using a trial-and-error method. However, the implementation of Monte Carlo Simulation could be an appealing alternative in this regard. There is much more work to be carried out in the research of calving time prediction and finding the optimal time for moving cows to calving pens.

## Figures and Tables

**Table 1:** Augmented Markov Chain Model.

States from/to	Transient State	Absorbing State
**Transient state**	*Q*	*R*
**Absorbing state**	*O*	*I*

## Appendix A

**Table A1:** Sample simulated activities data set (24 h).

Hours	Feeding	Moving	Lying	Standing	Stand Rumination	Lie Rumination
0–1	5.42	18.33	17.5	6.67	8.33	3.75
1–2	7.92	11.25	17.92	5.42	8.33	9.32
2–3	6.25	17.92	14.58	7.92	7.5	5.42
3–4	6.67	14.67	16.53	7.92	8.33	6.25
4–5	4.58	17.92	16.25	6.67	5.83	8.75
5–6	7.92	16.67	12.5	6.67	7.5	8.75
6–7	6.67	12.92	19.58	7.92	7.5	5.42
7–8	4.58	13.75	21.67	8.33	6.25	5.42
8–9	2.5	17.08	15.42	9.17	8.75	7.08
9–10	4.17	16.67	14.58	9.58	10	5
10–11	4.58	11.67	23.75	7.5	5.42	7.08
11–12	3.75	12.92	20.42	9.58	7.92	5.42
12–13	4.17	14.17	19.58	9.58	9.58	2.92
13–14	4.17	16.25	17.5	8.75	7.5	5.83
14–15	7.5	9.58	20.83	7.08	6.67	8.33
15–16	3.75	11.67	17.5	7.92	9.17	10
16–17	4.17	16.25	17.92	5	8.33	8.33
17–18	2.92	13.75	18.75	7.92	6.67	10
18–19	4.17	19.58	16.67	5.42	7.5	6.67
19–20	3.75	15.83	18.75	4.58	7.5	9.58
20–21	5	10.42	16.25	9.17	11.25	7.92
21–22	4.58	15.42	16.67	9.58	9.58	4.17
22–23	4.17	17.92	17.5	5	7.5	7.92
23–24	5	11.25	18.75	10	8.75	6.25

(A1)
$$C=\left[\begin{array}{cccccc} 6 & 3 & 1 & 1 & 1 & 1\\ 4 & 1 & 1 & 1 & 1 & 0\\ 1 & 0 & 2 & 2 & 0 & 2\\ 1 & 1 & 0 & 4 & 4 & 3\\ 0 & 1 & 1 & 4 & 3 & 0\\ 1 & 1 & 2 & 1 & 0 & 4 \end{array}\right]$$

(A2)
$$P=\left[\begin{array}{cccccc} 0.42 & \begin{array}{c} 0.23 \end{array} & \begin{array}{c} 0.08 \end{array} & \begin{array}{c} 0.08 \end{array} & \begin{array}{c} 0.08 \end{array} & \begin{array}{c} 0.08 \end{array}\\ 0.5 & 0.22 & 0.22 & 0.22 & 0.22 & 0\\ \begin{array}{c} 0.14 \end{array} & 0 & \begin{array}{c} 0.29 \end{array} & \begin{array}{c} 0.29 \end{array} & 0 & \begin{array}{c} 0.29 \end{array}\\ \begin{array}{c} 0.08 \end{array} & \begin{array}{c} 0.08 \end{array} & 0 & \begin{array}{c} 0.31 \end{array} & \begin{array}{c} 0.31 \end{array} & \begin{array}{c} 0.21 \end{array}\\ 0 & 0.11 & 0.11 & 0.44 & 0.33 & 0\\ 0.11 & 0.11 & 0.22 & 0.11 & 0 & 0.44 \end{array}\right]$$

(A3)
$$P^{\prime }=\begin{array}{cc} & \begin{array}{cccccc} \begin{array}{cc} s_{1} & \end{array} & s_{2} & \begin{array}{cc} & \begin{array}{cc} s_{3} & \end{array} \end{array} & s_{4} & \begin{array}{c} s_{5} \end{array} & \begin{array}{cc} \begin{array}{ccc} & s_{6} & \end{array} & s_{7} \end{array} \end{array}\\ \begin{array}{c} s_{1}\\ s_{2}\\ s_{3}\\ s_{4}\\ s_{5}\\ \begin{array}{c} s_{6}\\ s_{7} \end{array} \end{array} & \left[\begin{array}{cccccc} 0.27 & \begin{array}{c} 0.07 \end{array} & \begin{array}{c} 0.2 \end{array} & \begin{array}{c} 0.13 \end{array} & \begin{array}{c} 0.13 \end{array} & \begin{array}{c} \begin{array}{cc} 0.13 & 0.07 \end{array} \end{array}\\ 0.14 & 0.14 & 0 & 0.29 & 0.14 & \begin{array}{cc} 0.14 & 0.14 \end{array}\\ 0 & 0.33 & \begin{array}{c} 0.33 \end{array} & \begin{array}{c} 0.17 \end{array} & 0 & \begin{array}{c} \begin{array}{cc} 0.17 & 0 \end{array} \end{array}\\ \begin{array}{c} 0.29 \end{array} & \begin{array}{c} 0 \end{array} & 0 & \begin{array}{c} 0.43 \end{array} & \begin{array}{c} 0.14 \end{array} & \begin{array}{c} \begin{array}{cc} 0.07 & 0 \end{array} \end{array}\\ \begin{array}{c} 0.1 \end{array} & \begin{array}{c} 0.1 \end{array} & 0.1 & \begin{array}{c} 0.3 \end{array} & \begin{array}{c} 0.4 \end{array} & \begin{array}{c} \begin{array}{cc} 0 & 0 \end{array} \end{array}\\ \begin{array}{c} 0.44\\ 0 \end{array} & \begin{array}{c} 0\\ 0 \end{array} & \begin{array}{c} 0.11\\ 0 \end{array} & \begin{array}{c} 0\\ 0 \end{array} & \begin{array}{c} 0\\ 0 \end{array} & \begin{array}{cc} \begin{array}{c} 0.44\\ 0 \end{array} & \begin{array}{c} 0\\ 1 \end{array} \end{array} \end{array}\right] \end{array}$$

(A4)
$$Q=\begin{array}{cc} & \begin{array}{cccccc} \begin{array}{c} \begin{array}{cc} s_{1} & \end{array} \end{array} & \begin{array}{c} \begin{array}{cc} s_{2} & \end{array} \end{array} & \begin{array}{c} \begin{array}{cc} s_{3} & \end{array} \end{array} & \begin{array}{c} \begin{array}{cc} s_{4} & \end{array} \end{array} & \begin{array}{c} \begin{array}{cc} s_{5} & \end{array} \end{array} & \begin{array}{c} \begin{array}{cc} s_{6} & \end{array} \end{array} \end{array}\\ \begin{array}{c} s_{1}\\ s_{2}\\ s_{3}\\ s_{4}\\ s_{5}\\ s_{6} \end{array} & \left[\begin{array}{cccccc} 0.27 & \begin{array}{c} 0.07 \end{array} & \begin{array}{c} 0.2 \end{array} & \begin{array}{c} 0.13 \end{array} & \begin{array}{c} 0.13 \end{array} & \begin{array}{c} 0.13 \end{array}\\ 0.14 & 0.14 & 0 & 0.29 & 0.14 & 0.14\\ 0 & 0.33 & \begin{array}{c} 0.33 \end{array} & \begin{array}{c} 0.17 \end{array} & 0 & \begin{array}{c} 0.17 \end{array}\\ \begin{array}{c} 0.29 \end{array} & \begin{array}{c} 0 \end{array} & 0 & \begin{array}{c} 0.43 \end{array} & \begin{array}{c} 0.14 \end{array} & \begin{array}{c} 0.07 \end{array}\\ 0.1 & 0.1 & 0.1 & 0.3 & 0.4 & 0\\ 0.44 & 0 & 0.11 & 0 & 0 & 0.44 \end{array}\right] \end{array}$$

(A5)
$$R=\begin{array}{cc} & s_{7}\\ \begin{array}{c} s_{1}\\ s_{2}\\ s_{3}\\ s_{4}\\ s_{5}\\ s_{6} \end{array} & \left[\begin{array}{c} 0.07\\ 0.14\\ 0\\ 0\\ 0\\ 0 \end{array}\right] \end{array}$$

(A6)
$$N=\left[\begin{array}{cccccc} 8.14 & 3.2 & 3.88 & 6.84 & 4.2 & 4.82\\ 6.52 & 3.96 & 3.28 & 6.53 & 3.95 & 4.41\\ 7.19 & 3.64 & 5.14 & 7.19 & 4.18 & 5.13\\ 7.76 & 3.38 & 3.86 & 8.81 & 4.63 & 5.02\\ 7.52 & 3.49 & 3.98 & 7.83 & 6.03 & 4.9\\ 7.95 & 3.29 & 4.13 & 6.91 & 4.2 & 6.68 \end{array}\right]$$

(A7)
$$N=\left[\begin{array}{cccccc} 8.75 & 4.34 & 4.14 & 7.49 & 5.19 & 6.19\\ 6.92 & 4.79 & 3.74 & 6.81 & 4.74 & 5.47\\ 6.21 & 3.5 & 4.89 & 6.82 & 4.45 & 5.89\\ 7.2 & 4.24 & 4.23 & 9.27 & 5.82 & 6.75\\ 6.99 & 4.21 & 4.26 & 8.45 & 6.91 & 6.4\\ 7.95 & 4.07 & 4.38 & 7.44 & 4.93 & 7.84 \end{array}\right]$$
